# Sexual Dimorphisms in Innate Immunity and Responses to Infection in *Drosophila melanogaster*

**DOI:** 10.3389/fimmu.2019.03075

**Published:** 2020-01-31

**Authors:** Rebecca L. Belmonte, Mary-Kate Corbally, David F. Duneau, Jennifer C. Regan

**Affiliations:** ^1^Institute of Immunology & Infection Research, University of Edinburgh, Edinburgh, United Kingdom; ^2^Laboratoire Evolution & Diversite Biologique, UMR5174 EDB, CNRS, Université Toulouse 3 Paul Sabatier, Toulouse, France

**Keywords:** *Drosophila*, *Drosophila melanogaster*, innate immunity, sex dimorphism, aging, response to infection, sexual antagonism

## Abstract

The sexes show profound differences in responses to infection and the development of autoimmunity. Dimorphisms in immune responses are ubiquitous across taxa, from arthropods to vertebrates. *Drosophila melanogaster* shows strong sex dimorphisms in immune system responses at baseline, upon pathogenic challenge, and over aging. We have performed an exhaustive survey of peer-reviewed literature on *Drosophila* immunity, and present a database of publications indicating the sex(es) analyzed in each study. While we found a growing interest in the community in adult immunity and in reporting both sexes, the main body of work in this field uses only one sex, or does not stratify by sex. We synthesize evidence for sexually dimorphic responses to bacterial, viral, and fungal infections. Dimorphisms may be mediated by distinct immune compartments, and we review work on sex differences in behavioral, epithelial, cellular, and systemic (fat body-mediated) immunity. Emerging work on sexually dimorphic aging of immune tissues, immune senescence, and inflammation are examined. We consider evolutionary drivers for sex differences in immune investment, highlight the features of *Drosophila* biology that make it particularly amenable to studies of immune dimorphisms, and discuss areas for future exploration.

## Introduction

Sex governs physiology: differences between males and females are strong drivers of variance in phenotype within any population, and can eclipse effects of geography or genotype (1, 2). The immune system is no exception. Sex differences in human immunity are profound, where men and women respond differently to infection, treatment, diseases such as sepsis, and have different propensities toward autoimmunity (3, 4). However, the mechanisms underpinning these dimorphisms are largely unresolved. A major reason for this lack of resolution is that sex differences in immunity are understudied; in particular, there is a paucity of truly comparative studies. A recent meta-analysis addressing the issue of sex as a variable in biomedical studies showed that immunology as a discipline is particularly negligent, with fewer than 10% of studies reporting, or stratifying, by sex (5). Historically, women have been excluded from clinical trials and young males presented as “the norm,” in part due to concerns for potential impacts on fetal health (6). Parity has not been reached in representation (7) or reporting (8) of the sexes, despite the effective ban on women participating in clinical trials ending in the 1980s (6). In addition, in studies using laboratory model organisms, practical and budgetary considerations have led to the common practice of using a single sex. Recently, there has been recognition of the loss of knowledge propagated by the lack of inclusion of both sexes, with a drive from the scientific community to address the “gender gap,” including NIH and ERC commitments to address this specifically (9, 10).

The effects of immune dimorphisms are not only a consideration for clinical research, but also impact our broader understanding of host-pathogen interactions. Sex differences in immunity are observed throughout taxa, and are both cause and consequence of sex differences in life history, and sexual conflict. Responses to infection influence survival and fecundity, and therefore immune dimorphisms have the potential to affect both horizontal and vertical disease transmission throughout the animal kingdom (11). Inherant in the consideration of sex and immunity is complexity: within a single species, dimorphisms themselves are pathogen-specific (12), can respond to environmental variables such as diet (13, 14), and may even be influenced by the infective parasite which can be differently adapted to each sex (15). Adding further complexity is the interaction of immunity and sex with organism age (16).

As is the case for all insects, *Drosophila melanogaster* physiology is sexually dimorphic (17–19), yet despite its use for more than a century as a model organism, the extent of these dimorphisms are only just being fully appreciated (20). Sex differences are seen in immune tissues (21, 22) and in responses to infection (23), yet relatively few studies include both sexes. Studies that explicitly compare immune responses in both sexes in *Drosophila* reflect what is seen in other taxa in terms of prevalence and complexity: dimorphic responses are the norm rather than the exception, the direction that dimorphisms take with respect to the opposite sex is both pathogen- and context-dependent (23), and sex differences at baseline are not necessarily predictive of survival outcome (24).

### What Can *Drosophila* Teach Us About Immune Dimorphism?

*Drosophila* species have been used for many decades to study sexual antagonism in the evolutionary ecology field (25–27). We argue that including, and comparing, both sexes in functional and mechanistic studies of *Drosophila* immunity will add to this body of work to give important insight to several fields, in addition to better understanding host-pathogen interactions from an evolutionary ecology perspective. It will, for example, offer translatable information on disease vector biology, where sex is a crucial variable for exposure, transmission, and control strategies of insect disease vectors such as mosquitos (28–30). *Drosophila* provides a tractable model for innate immunity in mammals, as has been amply demonstrated over recent decades (31): studies on *Drosophila* could help understand rules underpinning sexual dimorphism in mammalian immunity and response to infections. Sex differences in mammalian immunity are often attributed solely to the action of steroid hormones. Interactions between steroid hormones and the immune system have also been demonstrated in *Drosophila* (32, 33), which may parallel endocrine-immune interactions in mammals. Mammalian immune dimorphisms arise not only as a consequence of selective pressures on the endocrine system. A large body of studies in mammalian immunology has uncovered many dimorphisms, particularly in autoimmune disease etiology, that are regulated by karyotype, independent of hormonal action (34–36). While it is difficult to attribute autoimmunity to organisms without immune self-recognition, direct self-damage by immune responses on the host has been demonstrated in *Drosophila* (37, 38) and may differ depending on the sex (39). *Drosophila*, like mammals, bear X and Y sex chromosomes, and both X- and Y-linked variation in immune responses have been demonstrated (40, 41). This, in combination with the strong conservation of immune signaling pathways (as exemplified by Toll/TLRs), makes *Drosophila* a powerful model for sex-specific genetic regulation of molecular immunity (23, 42).

### A Survey of Immunity Studies Using Adult *Drosophila melanogaster*

**Figure 1:**
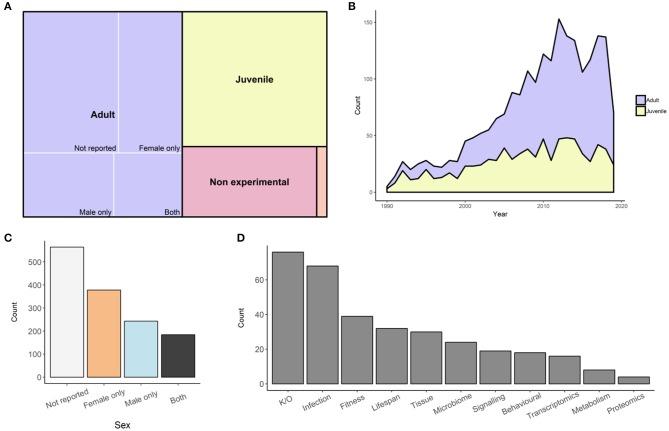
Composition of 2,614 articles on *Drosophila* immunity. **(A)** Categories used for all *Drosophila* immunity studies. Size of each rectangle is proportional to the number of articles within that category. The largest proportion are adult studies (purple; 1,366), followed by juvenile (yellow; 817), non-experimental (pink; 396), and bioinformatic (orange; 30) studies. **(B)** The quantity of articles published since 1990 until 21 August 2019 that use either adult (purple) or juvenile (yellow) *Drosophila*. **(C)** Quantity of adult *Drosophila* studies that do not report sex used (purple; 561), use females (pink; 378), use males (orange; 243), or use both sexes (yellow; 184). **(D)** Total number of articles that used both sexes, tagged according to experimental output. Tags are not exclusive, so some articles may have more than one tag. The tags used were gene function knock out (46), survival infection dynamics (47), fitness (39), lifespan (32), tissue specific (30), microbiome (24), signaling (19), behavioral (18), transcriptomics (16), metabolism composition (8), and proteomics (4).

We have undertaken to perform a survey of peer-reviewed, published studies of *D. melanogaster* immunity, reporting on the representation of sex within each study. We have focussed on studies using adult flies, while also identifying papers that use juveniles, cell cultures, or pre-existing genetic data. We searched for *Drosophila* immunity papers through Web of Science, attempting to avoid studies of other model organisms that mentioned *Drosophila*. As of the 21 August 2019, we downloaded the citations for the resulting 5,626 publications and manually categorized each paper. Two thousand eight hundred and forty-eight papers were removed since *Drosophila* were not used in the study, or the focus of the study was not immunity. We also made a decision to exclude the 166 endosymbiont studies that used both sexes from our analysis. There were also three papers which we could not access, which were removed (43–45). Of the remaining 2,614 papers, 1,369 used adults, 817 used juveniles, 396 were non-experimental (i.e., reviews or methods), and 30 were bioinformatics studies (Figure 1A). In the last 30 years, we can see how the *Drosophila* immunity field has grown and the relative use of adults or juveniles has changed. In 1990, only two adult studies and three juvenile studies were published (Figure 1B). Initially, the majority of studies were conducted on juveniles, but by 2010, the trend had reversed, where 75 studies used adults and 47 used juveniles. The use of adult *Drosophila* to study immunity has continued to grow, averaging over 100 adult studies per year between 2012 and 2018 (Figure 1B). Of the adult studies, 41% (564/1,369) did not report the sex, or did not stratify data by sex. When sex was reported, 45% used only one sex, with more studies using females (28%; 378/1,366), than males (18%; 243/1,366). Only 13% (184/1,366) of all adult studies reported results for both males and females (“both” category; Figure 1C). We additionally tagged each paper in the “both” category with the study type or data output (Figure 1D, Figure S1). Tags were not exclusive, where studies could be assigned multiple tags, resulting in more tags than articles in the output. The two most common tags were “gene function/knockout” (23%; 76/334), followed by “survival/infection dynamics” (20%; 68/334). The survey information is available as a searchable table, intended as a resource for locating data on immunity in both sexes (Table S1). Integrating information from this wealth of published data, we review what is reported about sex dimorphisms in immunity in *Drosophila*.

## Sex Differences in Infection Outcomes

### Survival and Pathology

*Drosophila* exhibit dimorphic survival and pathology in response to bacterial, viral, and fungal infections. Importantly, dimorphic survival and prevalence of infection are pathogen- and context-specific (Table 1).

**Table 1:** Reported sex biases in survival to infection by specific pathogens.

**Class**	**Pathogen**	**Survival bias-direction**	**References**
Viral	*Kalithea*	Female	(48)
Fungal	*Beauveria bassiana*	Male	(49–53)
	*Metarhizium anisopliae*	Male	(54)
	*Candida albicans*	Female	(55)
Microsporidial	*Tubulinosema ratisbonensis*	Female	(56)
Bacterial gram-negative	*Pseudomonas aeruginosa*	Male	(46)
	*Pseudomonas aeruginosa*	Female	(55)
	*Pseudomonas fluorescens*	Female	(22)
	*Providencia rettgeri*	Male	(23)
	*Providencia alcalifaciens*	Male	(23)
	*Coxiella burnetii*	–	(57)
	*Serratia marcescens*	Female (Genotype-specific)	(58)
Gram-positive	*Enterococcus faecalis*	Male	(23)
	*Lactococcus lactis*	Female	(58)

#### Viral Infection

Few studies have compared male and female responses to viral infection in *D. melanogaster*. Males are possibly more susceptible to acute viral infection: they demonstrate lower survival to the recently-described, DNA virus *Kalithea* (KV), which was isolated from infected individuals caught from a wild population (48). Males are also more susceptible to higher viral titers of the RNA virus *Drosophila C* (DCV) (59). Notably, dimorphism in survival to viral infection might be influenced by coinfection with *Wolbachia*, an endosymbiont providing viral protection (60). Indeed, interactions between DCV and *Wolbachia* infection status and sex have been observed in analyses of behavioral responses (61), discussed in more detail below.

Other effects of viral infection can impact the sexes differently, in addition to survival. For example, although females infected with KV generally survive, they suffer from ovary degeneration and a strong reduction in fecundity (48). Viral-induced female infertility is known to occur in infections with flock house virus (FHV) due to oocyte destruction (62). Thus, conclusions on sex-specific impacts of viral infection on fitness need to consider all consequences of infection, not just survival.

#### Viral Transmission

A recent study demonstrated that male-biased DCV titers are accompanied by higher levels of fecal shedding (59), which is in apparent contrast to an earlier study that found females to be better transmitters of DCV than males (63). It is not known whether load, or rates of shedding, are necessarily predictive of the ability of each sex to transmit viral infection; this may be dependent upon several additional factors, including infection route and behavioral responses to infection. Further studies correlating viral load and shedding with the ability to transmit infection in both sexes will be informative. Vertically-transmitted viruses by definition interact with host sex, given their route through infected gonads. For example, Sigma virus (*Rhabdoviridae*), a negative-stranded RNA virus, is transmitted vertically through the sperm or ovules. Male transmission of Sigma virus is required for persistence in the population, while transmission efficiency is higher for females than males (64). Infection with Sigma virus leads to sexually dimorphic gene induction, with more gene expression changes induced by infection in males than females (65). Infected females significantly upregulate structural chorion proteins, which could reflect manipulation by the virus to aid vertical transmission, or an ovary-specific defense response.

Given the very small number of studies addressing dimorphisms in survival to viral infections, we do not yet have the ability to make inferences about sex differences in anti-viral responses, nor indeed whether there are differences between responses to RNA and DNA viruses, or diverse viral species.

#### Fungal and Microsporidial Infection

**Figure 2:**
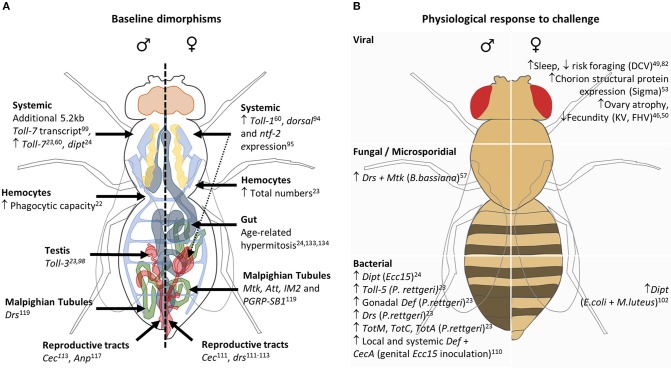
Schematic representing sexual dimorphisms in innate immunity at basal state **(A)** and in physiological responses to immune challenge **(B)**. **(A)** Male- and female-specific baseline conditions are depicted on the left and right of the central dashed line, respectively. Greater expression/ numbers are denoted by an upward-facing arrow, while stand-alone genes belonging to systemic or specific tissues represent sex-specific expression systemically or within that tissue, respectively. The dashed arrow represents the potential contribution made by the ovaries to observed differences in systemic transcript abundance. **(B)** Male (left column) and female (right column) physiological responses to viral, fungal/microsporidial, and bacterial are listed, with the causative pathogen in brackets. Increases and decreases in expression or behaviors are denoted by upward- and downward-facing arrows, respectively.

To our knowledge, there are only a small number of studies investigating sexual dimorphism in fungal infection. Indeed there is a dearth of studies investigating sex-specific physiological responses to such challenges (Figure 2). The most commonly studied fungal infection model, *Beauveria bassiana*, exhibits male-biased survival when flies are challenged via spore inoculation (49–53). The dimorphism appears to be, at least partly, attributable to dimorphic function of the Toll pathway, where loss-of-function mutants in Toll pathway components lose the sex difference in survival (52, 53). A similar male bias in survival is observed post-inoculation with the soil fungus *Metarhizium anisopliae* (54). However, although not directly compared, males appear to be more susceptible to systemic *Candida albicans* challenge by means of intra-thoracic injection (55). Interestingly, the effect of Toll-1 and Toll-7 mutation on resistance to *C. albicans* challenge revealed a greater sensitivity of male mutants to this fungal infection, while Toll-7 mutant females demonstrated resistance similar to that of controls (55). In addition, males succumbed to systemic infection with the microsporidium *Tubulinosema ratisbonensis* sooner than did females (56), despite a lower reported microsporidial load. However, the assertion made here that despite a greater lethality to *T. ratisbonensis*, males show a higher resistance as demonstrated by their lower pathogen load, needs further investigation. Pathogen load should be quantified before significant mortality has occurred in the population of infected individuals, otherwise, individuals with a high pathogen load who died prior to sampling would not be included in the analysis. A greater number of infected males had died 5 days post-infection from *T. ratisbonensis* than females, and pathogen load was analyzed 6 days post-infection (56). These results could potentially suggest that males are less tolerant, given that the only individuals still alive are those carrying a lower pathogen load.

Overall, in contrast to viral infections, males appear to survive longer than females in fungal infection models. There are indications that the magnitude of these dimorphisms in survival may be dependent on the environment. For example, the male-biased survival observed upon infection with *B. bassiana* is magnified by cold pretreatment of flies before infection, improving male survival at young and middle ages (81). Diet is also likely to influence susceptibility; for example, while females have decreased rates of survival to *B. bassiana* thoracic injection, immunity-induced metabolic declines were 50% greater in males (82). Supporting the interaction of metabolic state with sex-biased infection outcomes, polymorphisms associated with increased resistance to *M. anisopliae* inoculation are dimorphic and are biased toward gene networks regulating metabolism, as well as phagocytosis and cell migration (83). Furthermore, in the microsporidia infection model *T. ratisbonensis*, the quantity of circulating triglycerides was shown to affect parasite burden in females (56). The intersection of diet, metabolism and immune responses is likely to dictate outcomes to infection with most (or all) pathogens, as we discuss in more detail below. However, whether metabolic effects regulate resistance or tolerance to the fungal and microsporidial infection models described is as yet untested.

#### Bacterial Infection

The bacterial genus of *Wolbachia* and *Spiroplasma* have well-documented interactions with host sex and have been extensively studied in *Drosophila* infection models. Both bacteria disrupt the reproductive biology of their hosts, and show sex-specific transmission. While these interactions are entirely dependent on host sex, these examples are not strictly relevant to examination of dimorphic immune responses, and have already been extensively reviewed (47, 84–87), so we will not focus on them here.

In models of systemic bacterial infection of male and female adults, where immune responses and survival are directly compared, survival to infection appears to be pathogen-dependent. Depending on the amount and type of peptidoglycan (sugar- and amino acid-based polymers) in their cell wall, bacteria can be categorized into two groups, Gram-positive (G-) and Gram-negative (G-). In *Drosophila*, the production of immune effectors such as antimicrobial peptides is under the control of the immune deficiency (IMD) and the Toll signaling pathways. The former responds to the meso-diaminopimelic acid (DAP)-type peptidoglycan of G- bacteria and certain G+ bacilli, whereas the Toll signaling pathway responds mainly to the lysine (Lys)-type peptidoglycan of G+ bacteria and fungal beta-1-3-glucan (88, 89). Activation of immune signaling pathways are not strictly dictated by cell wall type, however; for example, both Toll and IMD signaling pathways are activated by *Staphylococcus aureus* infection (90).

It is not possible to neatly attribute the direction of sex-biased survival to bacterial infection to one particular signaling pathway. Males appeared to be more resistant to systemic infections by the extracellular G- *Providencia* species *Providencia rettgeri* and *Providencia alcalifaciens* (23, 91). However, mortality to the obligate intracellular *Coxiella burnetii* is comparable in males and females (57), while males died more quickly than females when infected with *Pseudomonas fluorescens* (22), or the extracellular bacteria *Serratia marcescens* (although notably, this was genotype-specific) (58). Few studies have assessed the response of both sexes to G+ bacteria. Females were reported to be more susceptible to infection with *Enterococcus faecalis* but less to *S. aureus* (23) or to *Lactococcus lactis* (58). Thus far, laboratory models of bacterial infection have demonstrated both male- and female-biased survival with G- and G+ species (Table 1). Different laboratories have also reported opposite biases in response to the same pathogen. For example, *Pseudomonas aeruginosa* is reported to induce dimorphic survival that is either male- or female-biased (46, 55), or not significantly different (22). These contrasting results may be dictated by genotype, as is evidently the case for male-biased susceptibility to *S. marcescens*, which was observed in only two out of four genetic backgrounds tested in a recent study (58). These results may also be influenced by environmental conditions such as mating status, which appears to have an immunosuppressive effect on females (23, 92, 93). Another probable reason a clear pattern has not emerged is that the bacterial species used in these infection models are more diverse than the peptidoglycan dichotomy belies. Compared to *P. rettgeri, S. marcescens* is highly pathogenic to *Drosophila*, and its infection dynamic within the host is very different (23). Moreover, while these two bacteria are extracellular, other species, such as *Coxiella*, infect intracellularly, further complicating the comparison. Different pathogens require different host defense mechanisms, which may rely on particular immune tissues, signaling pathways, and terminal effectors such as AMPs. A more systematic comparison between male and female responses to a range of different bacteria with different modes of infection will help to decipher the role for immune signaling pathways in dimorphism.

A role for the Toll pathway in mediating sex differences in survival to some bacterial infections has recently started to emerge. It appears to be necessary for dimorphic survival to *P. rettgeri*, and loss of Toll signaling reverses the survival bias to *E. faecalis* (23). Loss-of-function mutants of both Toll-1 and Toll-7 differentially affected infection outcomes in males and females. Toll-1 mutant males and females were less resistant to *E. faecalis* challenge than wild-type controls, while loss-of-function Toll-7 mutants reduced male resistance to both *E. faecalis* and *P. aeruginosa* (55).

### Behavioral Responses and Symptoms

In addition to dimorphic immune responses, *Drosophila* exhibit sex-specific behavioral symptoms and responses to infection. Grooming is thought to be an important behavioral defense against pathogenic infection, where flies remove potentially infectious microbes from their cuticle. Grooming in *D. melanogaster* is triggered by chemosensation of compounds, including pathogen components, by chemoreceptive sensilla. Specifically, the sensing receptor PRGP-LC contributes to grooming induction, connecting humoral immune sensing to behavioral responses (94). In optogenetic experiments targeting sensilla, grooming was more readily triggered in males than in females (94). Females were subsequently shown to rely more strongly on olfactory signals to remove cuticular *B. bassiana* than males, resulting in more conidia on the wings of olfactory-deficient female flies (95), but whether this contributes to the higher rate of survival by males after *B. bassiana* infection (49–53) remains to be seen. *Drosophila* exhibit dimorphic sleep responses to infection, a behavioral change which may be adaptive, or symptomatic. DCV causes females, but not males, to sleep more; however, DCV-infected male flies carrying *Wolbachia* are more lethargic when awake (61). Many other behaviors are likely to be impacted by symptoms such as lethargy and sleep alterations, including evasive behaviors and mating. For example, *Wolbachia* infection increases the recapture rate of females, but not males (96). Females previously exposed to DCV showed lower motivation to pick a food source when presented with a risk of encountering DCV (78). When disrupted, *nemuri*, an antimicrobial peptide that promotes sleep in *D. melanogaster*, reduced day-time sleep consolidation selectively in males (97), potentially linking dimorphic sleep behavior and responses to immune challenge. During a *P. rettgeri* systemic infection, females arrested egg-laying during the acute phase of the infection, until it stabilized into a chronic phase (98). The same observation has been made upon benign infections with *Pectobacterium carotovorum* (previously named *Erwinia carotovora carotovora*, or *Ecc15*) and *Escherichia coli* (99). The adaptive role of this behavioral response (99, 100) is not clear and is by definition female-specific. It remains to be determined if changes to reproductive behavior occur in males. Although research in this area is in its infancy, these studies suggest behavioral dimorphisms in *D. melanogaster* could be an important driver for sex differences in infection outcome.

## Sex Differences in Immune Compartment Physiology

Sex differences in outcome to infection are likely to be mediated by distinct immune compartments, where the key tissues involved will be dependent on pathogen and route of infection. Below we discuss those studies that assess the contribution of individual immune tissues. Sexually dimorphic immune physiologies in unchallenged flies and after acute infection are summarized in Figure 2.

### Hemocytes

Hemocytes are specialized immune cells responsible for the encapsulation and phagocytosis of pathogens and dead cells. Few studies focusing on *D. melanogaster* hemocytes have reported the sex of individuals used; but those that have, present some evidence suggestive of a dimorphism in this branch of the immune system. First, it appears that hemocytes can have sex-specific functions. The Jun N-terminal kinase (JNK) signaling pathway regulates the decision between cell repair and cell death. As such, *JNK*/*Basket* is required in larval hemocytes to promote tissue maintenance, but only in males, such that hemocyte-specific loss of *JNK*/*Basket* results in increased tissue damage in males after UV irradiation (101). In addition to this functional difference, there is some evidence for sex differences in total hemocyte number, but neither the direction of bias nor the drivers for the dimorphism can yet be concluded. Female white prepupae have been reported to contain a higher total number of hemocytes than males (102). In adults, higher numbers of hemocytes *per se* in females (23), and higher numbers of hemocytes per unit of hemolymph in males (21), have been reported, whilst a recent study found no effect of sex on adult hemocyte number (22). A subtly higher phagocytic index has been reported for male hemocytes *ex vivo* (22); however, sex differences in the functional roles of hemocytes in homeostasis or responses to infection are as yet unknown.

### Melanisation

Melanogenesis is an important feature of arthropod physiology. In addition to its role in cuticular hardening, via the synthesis of a specific melanin called sclerotin, and in cuticular coloration, via the synthesis of eumelanin, multiple studies have demonstrated its role in immune responses [see (103) for review]. An essential role for immune melanization in the efficient killing of encapsulated parasitoid wasp eggs and pathogens via cytotoxicity of reactive oxygen species (ROS) produced by the melanization cascade is well-appreciated (104, 105). The process hinges on cascades of serine proteases (SPs) triggered by either direct or indirect antigenic recognition and tissue damage (106). A key enzyme in this process is phenoloxidase (PO), which mediates the oxidation of the amino acid tyrosine to dihydroxyphenylalanine (DOPA) and subsequently, the oxidation of DOPA and dopamine to their respective quinones which are precursors of brown/black eumelanin. PO is produced as prophenoloxidase (PPO) proenzyme which is converted to active PO by a clip domain serine proteinase. This cleavage generates ROS, giving immune melanization its cytotoxic activity.

Whereas the biochemical pathways downstream of PO are well-characterized, our knowledge of the molecular events leading to PPO activation are largely unelucidated (103). The involvement of Toll signaling in melanization responses has recently been demonstrated in adults (106). Hayan and SP7, two SPs acting upstream of Spätzle, activate PPO1 and PPO2, which were shown to be essential for effective resistance against several systemic fungal and G+ bacterial challenges (106, 107). Mutants for *PPO* lack melanization, yet, maintained a dimorphic survival to *P. rettgeri* infection (23), suggesting that the activation of this immune response is not a driver of sex differences in survival to this particular pathogen. However, basal expression of some genes involved in melanogenesis are higher in males and a subset of these, including *Dopa decarboxylase (Ddc)* and *yellow-f*, respond transcriptionally to *P. rettgeri* infection (23). Alternative infection models where melanization is required to effectively control the infection may be more informative for understanding its potential roles in immune dimorphism. For example, the response of males and females to parasitoid wasp infection at larval stages has not been reported to our knowledge. Despite the importance of melanization for resistance to infection and injury, and the intersection of immunologic melanin production and Toll signaling, virtually no studies have assessed the sex-specific physiology of the response. Given that constitutive expression of Toll pathway genes, namely components upstream of Toll-1, as well as *Ddc*, is greater in males (23), the hypothesis that males are more poised for melanization warrants testing.

### Systemic (Humoural) Immunity

A number of studies have reported sex differences in systemic immunity (Figure 2). Systemic immunity is primarily driven by the fat body; however, expression levels of pathway components were largely measured in whole individuals. For example, following systemic challenge with *P. rettgeri*, males induced a number of Imd- and Toll-regulated effectors at higher level than females, paralleling their greater survivorship (23). Although to note, ablation of the Imd pathway does not suppress the sexual dimorphism in survival (23). Sex differences have repeatedly been found in expression and function of Toll pathway components. Females are reported to exhibit higher expression of Toll-1, the transmembrane receptor that activates the Toll intracellular signaling pathway (55), the Relish protein *dorsal* (74) and the Toll pathway component *ntf-2* (75). The Toll pathway is known to be involved in two processes: dorso-ventral embryonic patterning, a female specific process occurring in the eggs, and in immunity. Signaling downstream of the transmembrane receptor Toll-1 is shared between these two processes. Thus, it is unsurprising that the apparent dimorphism in Toll pathway gene expression disappears once expression in the ovaries is excluded (23). Nonetheless, the expression of the Toll pathway seems crucial for the sexual dimorphism of many infections. This includes both G+ bacterial infection but, perhaps surprisingly, also G- bacterial infection (23). This occurs through the activation of the pathway by *Persephone*, a hemolymphatic serine protease, which senses microbial proteases during infection (108, 109). Toll-3, also called MstProx, shows male-specific expression and response to infection, which seems to be attributable to the gonads (23, 72). Toll-5, or *Tehao*, is also induced at higher levels in males than females following *P. rettgeri* infection (23). Toll-7, a plasma membrane Toll receptor that binds to viral glycoproteins, is expressed at higher basal levels in males (23, 55) and appears to have an additional, male-specific isoform (73).

Male-biased survival to *B. bassiana* also appears to be dependent on the Toll pathway, since mutations in Toll pathway components ablate or reverse the dimorphism (53). The induction of *drosomycin* and *metchnikowin* in the first 24 h of infection also tended to be greater in males (52). The survival dimorphism, however, was not suppressed when tested on *spaetzle* (*spz*) mutants, a component of the Toll pathway (52). These Toll-regulated dimorphisms may in part be mediated by expression of AMPs: for example, *attacins* and *diptericins* require functional Toll for full inducibility. Supporting this, the loss of Toll affects the *Enterobacter cloacae*-induced expression of *attacins* and *diptericins* more strongly in males (110), and males with a gain-of-function Toll mutation exhibit higher levels of the AMPs *Cecropin A1 (CecA1), Diptericin A* (*DptA), Attacin A (AttA)*, and *Attacin B (AttB)* in response to challenge (111). Furthermore, dimorphic induction of *drosomycin* in response to *P. rettgeri* infection is lost in *spz* mutants (23). Dimorphisms have also been reported in Imd pathway components, for example, several Imd-induced AMPs are found to be dimorphic in their expression levels. Using a construct with various *diptericin* promoter sequences upstream of the *lacZ* gene, higher levels of induction were seen in females than males (79). In contrast, *diptericin* has been shown to be expressed at higher levels in unchallenged males than females, and is more strongly upregulated upon infection with the G- bacterium, *P. carotovorum* (24).

The JAnus Kinase protein and the Signal Transducer and Activator of Transcription (JAK-STAT) pathway, required for antiviral immune responses and induced upon bacterial infection, also shows dimorphic expression. *G9a*, a histone H3 lysine 9 methyltransferase, regulates tolerance to viral infection by regulating JAK/STAT (112) but in a sex-specific manner, with females being more sensitive to a loss of *G9a* (113). In addition, the stress-responsive genes regulated by the Jak-Stat pathway, *TurandotA, TurandotC*, and *TurandotM*, were among the genes that were the most male-biased in an analysis of the transcriptional response to *P. rettgeri* infection (23), although the consequences of this are as yet unknown. Overall, males have a higher expression of many Toll- (and to a lesser extent Imd-) regulated genes. The reason for this dimorphism is unclear, but further work could investigate a potential link with the dual role for the Toll pathway in females. It is possible that the immune response via the Toll pathway in females is constrained by its consequences on egg development, a constraint which does not apply to males.

### Epithelial Immunity

Comprising the third prominent arm of defense in *D. melanogaster* are the immune-reactive epidermal and epithelial barriers such as the cuticle, trachea, genitalia, and gut.

#### Cuticle and Trachea

Sex differences in defense against fungal inoculation of the cuticle with the entomopathogenic *B. bassiana* demonstrate female-biased susceptibility (53, 114–116). Dimorphisms in cuticle integrity, or in immune responses of the cuticle or respiratory system could conceivably be underpinning this susceptibility. The trachea, which consist of airway epithelia and spiracles, is immunogenic, and responses may be activated by such cuticle inoculations. Studies that have assessed tracheal immunity in adults, thus far include only males (71, 117), rendering sex-specific responses within respiratory tissue another unexplored, potential contributor to dimorphic immunity. Dimorphisms in cuticular epithelial immune responses *per se* are also, to our knowledge, entirely unexplored.

#### Genitalia and Gonads

Male genitalia were found to be more primed for immune response to bacterial infection than that of females, where systemic and local AMP responses followed *P. carotovorum* inoculation of the genitalia in males (80). The female reproductive organs are also immune active, and were shown to constitutively express *cecropin* (66) and *drosomycin* (66–68), where *drosomycin* expression was found to be independent of Toll signaling in this tissue (67). Males exhibit constitutive expression of *cecropin* within the ejaculatory duct, independent of Relish signaling (68), suggesting that genital epithelia may circumvent classical pathways to activate AMPs. Immunogenicity of the reproductive tissues is evident from analyses of post-mating AMP responses in females (118, 119), and the antimicrobial protein transfer from male accessory gland and ejaculatory duct to the female (120). Andropin, an AMP unique to males, is also strongly upregulated in response to mating (69). RNA-seq analysis of *P. rettgeri* infected males and females, with and without gonads, illustrates the contribution made by reproductive tissue to systemic immune responses (23). Such comparative transcriptomics revealed male-biased *defensin* levels following infection to be gonad-dependent (23). It is an open question how much reproductive tissues contribute to systemic immunity; indeed it is unknown whether AMPs produced by the gonads are released into the hemolymph, or remain within the tissue.

#### Malpighian Tubules

Malpighian tubules (MT), epithelial organs dedicated to filtration and analogous to the mammalian kidney, are also immunogenic (121). Transcriptomic analysis of MT revealed differences in basal immune gene expression between the sexes (70); however, nothing is known about sexually dimorphic functions of MT or their contribution to immune dimorphisms.

#### The Intestinal Epithelium

The *Drosophila* gut is a major immune locus, responding to infection by producing AMPs and reactive oxygen species (122, 123). Infection-induced immune responses are observable in males (122); however, while a small number of studies have compared sex differences in gut metabolism (124), and physiology (24, 124–126), most work on intestinal immunity has focussed on females. Nothing is known about sex dimorphisms in AMP or ROS production, nor indeed survival, after oral infection. However, expression of several immune-related genes have been reported. *Nubbin*, a transcription factor with two isoforms, *nub-RB* and *nub-RD* (127) regulates duration of immune responses within the gut. *Nub-RD* mutants showed chronic immune activation in females (128), whilst overexpressing *nub-RB* resulted in a similar phenotype, illuminating their antagonistic roles within immune signaling (127). Overexpressing *nub-RB* within enterocytes and subsequent oral infection with *P. carotovorum* lead to total and 70% death in males and females, respectively, within 24 h. Downregulation of *nub-RB* enhanced survival to challenge in males compared to controls, while having the opposite effect on females (127). Overexpressing *nub-RB* significantly reduced the lifespans of both sexes, conversely, males reared in germ-free conditions had a slightly enhanced median longevity compared to conventionally-reared counterparts (127). These data could potentially illustrate a greater susceptibility of males to immunopathology from both chronic immune activation and commensals. *Nubbin* isoform antagonism may also offer insight into dimorphic responses between the sexes, where expression of each may differ, however, the cited study only quantified isoform expression in males (127).

The luminal microbiome impacts intestinal immunity (123) as well as many other aspects of *D. melanogaster* physiology and behavior (129). This includes cellular immunity (130), and response to enteric viral infection (131). Studies have shown that the microbiota varies between the sexes in terms of load (24), composition (132), and effect on metabolic responses to diet (133). However, most studies assessing the gut microbiome have only analyzed females, and thus we have very little knowledge about the interaction of commensals with dimorphisms in intestinal immunity. Microbiota populations, at least of the two predominant genera *Acetobacter* and *Lactobacillus*, tend to increase over aging (134), and composition varies between the sexes, where aged males were reported to differ to a greater extent than aged females when compared to their younger counterparts (132). Aging is also associated with a loss of epithelial barrier integrity in the gut (76, 77, 135). This phenomenon is more pronounced in females (24, 76), and is paralleled by increases in systemic immune activation, as indicated by systemic AMP levels (76). Microbiota dysbiosis (77) and loss of a tricellular junctional protein, Gliotactin (135), were shown to precede such changes in females, and loss of an intestinal septate junction protein, Snakeskin, exacerbates barrier loss and causes early death in both sexes (136). These data highlight the link between microbiome dysbiosis, maintenance of a stable gut barrier, and systemic inflammation. More work is needed to understand the interaction of the dimorphisms involved; for example, the apparently conflicting observations that the male microbiome shows greater changes (132), but despite this, the male gut barrier appears to be more stable over age (24). It is well-known that the microbiome is modeled by diet, environment, and host genotype, so comparing physiological data with microbiome data from different labs may confound interpretation.

### Aging and Immune Dimorphisms

#### Aging and Inflammation

Aging is known to be accompanied by heightened expression of immune genes (37, 39, 137–139). However, cause and consequence are difficult to separate, and understanding the contributions made by age to altered immunity, and immunity to aging, is challenging. When comparing the transcriptional response to aging in whole flies, a strong sex-by-age interaction was observed (139). Of the total significant probe sets, the majority were biased toward males, while just over 25% were sexually antagonistic. Included within this male-biased and antagonistic set were immune-related genes, such as the AMP, *defensin* (139).

In an experimental evolution study on lines where late-life fertility, and indirectly longevity, was selected for over 35 years, decreased expression of immune genes was strongly associated with increased lifespan (137). Females of longer-lived lines exhibited greater realized immunity in the face of challenge with *P. carotovorum, B. bassiana, E. faecalis*, and DCV than controls (137). Modulation of Toll components had varying effects on male and female lifespan: knockdown of the negative regulator *cactus* had a significantly reducing effect on lifespan, especially in males, whereas *Toll* and *spz* knockdown enhanced lifespan in both sexes (137). *Dif* knockdown had opposing outcomes between the sexes, with females experiencing a slight extension to lifespan (137). However, in two other studies, *Dif* mutants exhibited enhanced lifespan, in the context of intrinsically short-lived background lines (50, 140). Given the sex-specificity of Toll pathway gene expression seen in young flies (23), it is perhaps unsurprising that responses to such modulations are dimorphic.

Age-related systemic inflammation, for example, high basal levels of AMPs and ROS, has been assumed to negatively impact lifespan. This is supported by studies in which AMPs, or Rel-family transcription factors controlling their expression, have been manipulated. Both systemic and fat body-specific overexpression of *relish* negatively affected male lifespan to a greater extent than that of females, and *relish* male mutants were marginally longer-lived than females (39). The selective knockdown of *relish* at mid-life stages in the fat body, however, significantly extended lifespan in males (39). Global overexpression of *attacin A, cecropin A1, defensin*, and *metchnikowin* had significantly deleterious effects on lifespan of both sexes, with overexpression of *defensin* having a greater impact on males (39), reminiscent of the increase in *defensin* expression in aged males (139). This potentially illustrates age-related immunopathology, where the tempering of hyperactivated immune pathways at mid-late life stages is protective. Intriguingly, clean injury of the cuticle has been shown to extend lifespan in males only, suggesting that a non-lethal wound initiates a response that has a hormetic effect that is particularly effective in males (141). The mechanism for this is unknown, but it is tempting to speculate that it could initiate an anti-inflammatory state through induction of immune regulators.

#### Immunosenescence

Relatively few studies have looked at immune function in both sexes over aging, particularly from a mechanistic standpoint. While survival following *B. bassiana* decreases over aging in both sexes (140), systemic and cuticular inoculate challenge with the entomopathogenic fungus was reduced in aged females compared to young, while males succumbed only to cuticle inoculation, exhibiting a reduction solely in barrier integrity (114). Age negatively affected the ability to survive an oral *P. carotovorum* challenge in males compared to females, despite their comparatively superior maintenance of intestinal barrier integrity (24). In addition, aged males fared worse in response to systemic *E. coli* (142), *P. aeruginosa*, and *Bacillus thuringiensis* (22) challenges. The studies described above do not investigate the mechanisms underpinning these age-related immune dimorphisms, nor indeed the tissue(s) responsible, except via route of infection (24, 114). In a study investigating the effects of age on hemocyte function, hemocyte numbers were shown to decrease selectively in females over aging (21), supported by a recent study that only examined females (143); although both sexes maintained *ex vivo* phagocytic capacity over age (21). In contrast, a recent study found no effect of age on hemocyte number (22). It is clear that the mechanistic underpinnings of sex-by-age interactions in efficacy of immune responses and autoinflammation are undefined, but could be hugely informative to our understanding of sex differences in age-related pathology and lifespan.

## Evolution of Dimorphism Through Natural Selection

Males and females can have different life histories, which implies that they are exposed to different evolutionary pressures. Because the sexes share a genome, these different pressures can lead to sexual antagonism, which can potentially be resolved through the evolution of sex-specific regulation leading to phenotypic dimorphism. We explore this in the section below with reference to the immune system in *Drosophila*.

One possible driver of natural selection on dimorphism is unequal exposure to parasites in males and females. Males and females may differ in the habitats they occupy, their activity times, or their nutritional needs, for example. Males and females thus occupy separate niches and are potentially exposed to different parasites. Parasites can exert different selection pressures on males and females: the extent to which they do will depend on the extent of ecological divergence between the sexes. Knowledge of the ecology of wild *Drosophila* being relatively limited, it is difficult to define precisely which behaviors could cause such sex-specific selection. Females lay their eggs in rotting fruits, where they are necessarily exposed to a microbe-rich environment. Whether males spend less time in these environments is not known. Lab studies have indicated that male and female *Drosophila* do not have the same optimal diet, and in diet choice experiments, make different nutritional selections in accordance with their role in reproduction (144–146). Males and females could potentially make different diet choices in the wild, and thus be exposed to different parasites.

A second possible driver of natural selection on dimorphism comes from the fact that the immune system allows resistance to parasites but also represents a cost: it requires a significant investment in terms of resources, and the activation of immune defenses can cause “collateral” damage [e.g., autoimmune reactions (147)]. From this it is predicted that hosts will evolve toward an immune response of intermediate intensity, and not toward a maximum response (148). In other words, hosts evolve within a framework of constraints corresponding to an evolving trade-off. But the terms of this trade-off are not necessarily the same for both sexes: Hamilton and Zuk proposed that the links between investment in immunity and life history traits are sex-specific (149). Under this assumption, it can be predicted that the optimal investment in immunity is not the same in males and females, and could even be antagonistic (150). This scenario is supported by evidence in *Drosophila* that both resistance and tolerance can be sexually antagonistic (46), that expression of immune genes is sexually dimorphic (27), and that the genetic architecture of many traits, including immunity, differ between males and females in *Drosophila* (151–153). This is supported by mutation accumulation experiments indicating that deleterious mutations do not have the same costs in both sexes (154–156). Although the accumulation of spontaneous mutations in *Drosophila* has not yet been shown to have a sexually dimorphic cost on the immune response (46), it does appear have a sex-specific effect on fitness (156). If autosomal determination of immune traits are not clear-cut, the fact that *Drosophila* immune genes can be X- and Y-linked (40, 41, 157) is sufficient to expect that genetic structure can affect immune system evolution in a sex-specific manner. First, the Y chromosome is, by definition, strictly under selection in males and has been demonstrated to influence the immune response (41, 157). For example, only males that had a Y-chromosome introduced from a single wild population differed in their ability to defend against *S. marcescens* (41). Second, since fathers do not pass an X-chromosome to their sons, evolution by sexual selection acting on males would be much slower for traits largely influenced by the X chromosome than would selection on autosomally determined traits, and X-linked sexually antagonistic traits would more freely affect sexual dimorphism (158). Supporting this is the demonstration of X-linked variation in immune response phenotypes in *Drosophila*, including bacterial load and immune gene expression, in a set of 168 X-chromosome extraction lines (40). Many of the associations of genetic variation with immune phenotype acted in a sex-specific or sexually antagonistic manner, supporting the theory that sexually antagonistic variation may be more easily maintained on the X chromosome, and this can impact dimorphism (158).

### Sexual Selection and Evolution of Dimorphism: The Hypothesis of the Susceptible Male

Sexual selection is that which operates on the ability to mate successfully. Two traits that generally evolve in parallel in each sex under the effect of sexual selection are choice by females and the ornaments of males. Ornaments are expensive secondary sexual characteristics that evolve as a result of the selection made by the choice of females. A difficulty with this principle is that females can only choose males based on a trait that demonstrates their vigor if that trait remains variable. For sexual selection to occur, therefore, a continuous source of heritable genetic variation must be present in the population. This source of variation prevents genes for vigor from fixation and the character of choice to be only affected by non-heritable environmental factors. Parasites of all kinds offer such selective variation in value because they can evolve quickly and dynamically with their hosts (149, 159). It is on this basis that the famous hypothesis of William (Bill) Hamilton and Marlène Zuk was born. They proposed that parasite selection imposes the evolution of female choice for infection-resistant partners, which in turn favors the evolution of sexual dimorphism in hosts. Thus, as they observed in birds, a negative correlation between ornaments and parasitic load can be found in males (149). Such correlation between expensive ornamental traits and immunity may be present in *Drosophila*. One of the most clearly dimorphic characteristics in *Drosophila* is the color of the cuticle, which is darker in males than in females. The process of darkening the cuticle requires the production of eumelanin, which is also involved in the encapsulation of parasites. This dual role suggests that coloration and immunity are two related characteristics (103, 160). Furthermore, as mentioned above, melanin is produced from dopamine, a neurotransmitter also known to be involved in aggression behavior between males (161), male courtship for females (162) and female receptivity (163). Dopamine and melanin production are thus located at a metabolic crossroads establishing a link between secondary sexual characteristics, mating behavior, and immunity. Other visible, dimorphic characteristics in *D. melanogaster* include bristle number (abdominal and sternopleural), and the presence of sex combs, specialized leg bristles in males which aid copulation. Surprisingly, these traits have not been used, to our knowledge, to study sexual selection, despite being very clearly exposed to females. A recent study reported that flies with a mutation in the *yellow* gene, which encodes a protein involved in the synthesis of eumelanin, fail at mating because insufficient melanization renders their sex combs non-functional for grasping and mounting females during copulation (164). Furthermore, determination of bristle number in males is connected to the process of melanization (165). This link is confirmed by genetic analyses that have shown that *Ddc*, an enzyme essential for dopamine synthesis, has a role in pigmentation (166), and also in the determination of the number of bristles (165). Interestingly, *Ddc* is expressed differentially in males and females, as expected, but its expression is modulated during a bacterial infection [see RNA-seq data for “*Ddc*” in (23)] (167). A mutation in the *ddc* gene could therefore have an impact on both male ornamentation and response to infection. Female *Drosophila* are able to choose their mates on the basis of phenotypic traits (168, 169), and while evolutionary theory offers several different explanations for female mate choice, most include the evolution of heritable attractive features in males (170). The *Drosophila Genetic Reference Panel* (i.e., DGRP lines) show that natural populations indeed bear variation at several positions in the coding sequence of *Ddc* (see genome browser at genome.ucsc.edu). In *Drosophila*, it is unclear how male ornaments (or attractive traits) trade-off with investment in immunity, but one could speculate that factors required for both cuticle patterning and immune responses could be limiting. Since males have a high selection pressure for access to mating (25), they might still invest in their costly ornament (e.g., cuticle, bristles) to the detriment of their immunity. In any case, the hypothesis that investment in immune melanization may affect secondary sexual traits, such as bristles/combs, remains to be tested.

Hamilton and Zuk have shown that the dimorphism of investment in immunity increases with the intensity of sexual selection. This observation can be explained by a direct negative effect of reproductive traits on those of immunity: this is the so-called immunocompetence handicap hypothesis (171). It occurs because the immune system can interact directly with the hormonal system in relation to sexual dimorphism (172, 173). Increased production of hormones may thus have a benefit for secondary sexuality in males (e.g., ornaments, or competitive traits such as muscle mass) but a deleterious effect on the immune response. Since males have a high selection pressure for access to mating, they might invest in competitive traits to the detriment of their immunity (174). The handicap hypothesis implies that the resource allocation trade-offs between immunity and other characteristics related to selective value are not the same for males and females (148, 175, 176), and leads to the conclusion that males should invest fewer resources in immunity than females. In males, the benefits of increased mating success through greater investment in sexually selected traits (or in costly behaviors) should offset the costs of disease-related reduction in lifespan. Females, on the other hand, would invest more in immunity than males to maximize their breeding time. This prediction extends Bateman's principle to immunity (25, 177). In *Drosophila*, the interaction between immunity and hormones has clearly been identified (32, 33, 93, 178). For example, females produce juvenile hormone (JH) to lay fertilized eggs, and JH directly suppresses the immune response (93). JH affects the antimicrobial peptide (AMP) Drosomycin most strongly, which responds mainly to G+ bacterial infection via the Toll-mediated immune pathway (32). Interestingly, as discussed above, a greater susceptibility of females to some infections is mediated by Toll signaling. This is suggestive that the sex-specific investment of females in egg production via the production of JH has a cost on their immune system, leading to sexual dimorphism in response to certain infections (23).

The previous hypotheses suggest that one sex should be superior than the other while facing infection. Although these hypotheses predict dimorphism *per se*, they cannot explain the inconsistencies in the direction it takes. Alternatively, sex-specific differences may arise from sex-specific changes in reproductive behavior in response to variation in fitness-limiting resources availability, and not from an intrinsically superior immune function (179). In such a case, sex-specific responses would be condition dependent. In *D. melanogaster*, availability of sexually receptive females is an important fitness-limiting resource. While males adjust their level of courtship in response to this resource, increased sexual activity reduces their immune functions, likely because of a reallocation of feeding time into the search for a mate (180). Food availability is, of course, a major fitness-limiting resource. When males and females were kept separate and given *ad libitum* food, immune function was maximized in both sexes and there was no sexual dimorphism in clearance of a benign infection with *E. coli*. However, when the fitness-limiting resources were scarce there was a sex-specific bias (179). When food was limited, females exhibited poorer clearance of the infection than males and, conversely, males with high sexual activity, despite abundant food, performed less well than females. Thus, if there are many reasons for dimorphism to evolve, there are many reasons for its direction to be plastic.

### Obstacles to the Evolution of Dimorphism

If different selection pressures are applied to two subpopulations, there is every indication that they will evolve in different ways (181). However, males and females of a species are not sub-populations like any other, since their genomes must recombine in each generation. In each generation, therefore, a gene that is advantageous when expressed in a female can be transmitted from a mother to her son, in whom the expression of this gene could be disadvantageous. In summary, the evolution of a beneficial trait for one sex can be a burden for the other sex (182). In the previous example, the genetic origin of this burden is antagonistic pleiotropy: an allele beneficial to one sex has a negative effect on the other. This sexual conflict, highly investigated in *Drosophila*, can occur at one locus (intra-local conflict: a mutation at one locus has a positive effect for one sex but negative for the other) or at several (interlocal conflict: a mutation at one locus has a negative effect on one sex and selects a mutation at another locus that reduces the effectiveness of the first mutation) (26). This sexual antagonism could mean that the advantage obtained by one sex is exactly offset by the burden the other sex suffers. In other words, antagonistic pleiotropy could prevent the evolution of dimorphism. In a less extreme situation, if dimorphism manages to evolve despite pleiotropy, it is conceivable that neither sex will be able to adapt optimally.

### Dimorphism as a Resolution of Sexual Conflict

It is also possible that selection may favor sex-specific regulatory genes that result in an advantageous allele in females being expressed only in females (183). Pleiotropy would then be eliminated, and dimorphism could evolve without constraint. To our knowledge, there is no experimental work that has studied the role of this sexual conflict in the evolution of sex-specific regulation of the response to infections in *Drosophila*.

### One Pathogen, One Rule

It is usually impossible to predict, for a given infectious disease, which sex will suffer the most. For example, in mammals, it is often said that males are the “susceptible” sex (184). Men are indeed more susceptible to leishmaniasis, malaria, or bilharzia; but they are more resistant to toxoplasmosis, amebiasis, and giardiasis (12, 185, 186). The complexity increases considering that pathogens can adapt specifically to the sex-specific characteristics of the host. If a parasite is more often exposed to one sex, it is expected to adapt to that sex it encounters most frequently (187). Under such a scenario, even in the absence of sexual dimorphism in response to the infection, the parasite is expected to behave differently and to consequently induce a dimorphism in symptoms or virulence (15, 188). Attempts to generalize, through meta-analyses, are not consistent: they sometimes even imply that there is no real dimorphism (189). If the selection pressures applied to both sexes vary considerably from one parasite to another, we cannot exclude that the effects of the different parasites cancel each other out, so that the average selection pressures applied by all parasites ultimately lead to the same investment in immunity in males and females. Given that different parasites require different methods of control, this seems an unlikely outcome; instead, varied selection pressures, applied differently to each sex, requiring distinct responses, could result in a complex array of context-dependent dimorphisms. In *Drosophila*, reported directions of dimorphism in infection outcome are condition- and pathogen-dependent (Table 1), thus it seems inappropriate to propose a generality on the direction of immune sexual dimorphism (23, 40). However, even if the direction is difficult to predict, it is rare that dimorphism does not occur: in that sense, dimorphism in infection outcome seems to be the rule and not the exception.

### Environmental Effects on Immune Dimorphism

As if pathogen-dependent dimorphism was not complex enough, infection almost certainly interacts with environmental factors to shape immune dimorphisms. Nutrition modifies the response to infection (190, 191), and where choice is offered, flies will change their feeding preferences in response to infection (192). Diet choices have been empirically demonstrated to increase fitness in insects (193), e.g., in a “true fruit fly” (*Bactrocera tryoni*)—*S. marcescens* infection model, where infection-induced selection of increased carbohydrate-to-protein ratio led to better survival (194). Baseline and infection-induced sex differences in nutrient preference may influence outcome to infection, as may dimorphic responses to macronutrients. For example, in a *D. melanogaster*—*Vibrio cholerae* infection model, increased levels of glucose selectively reduced lifespan in females, but delayed their succumbing to infection, whereas it had no effect on males (195), suggesting that while females suffer long-term consequences of chronically elevated insulin signaling, acute responses to pathogenic challenge, at least by *V. cholerae*, are enhanced by high dietary glucose (195). Nutrient-sensing pathways are known to regulate immune responses, among many other physiological responses to the environment (196), and perturbations in these pathways are shown to affect sex-differential gene expression in *Drosophila*, including expression of immune, defense, and stress response genes (146, 197). Thus, it is essential that we view diet, nutrient-sensing, and sex as interacting factors when considering immune dimorphisms.

Other biotic (density, competition), and abiotic (temperature, humidity) factors are likely to play into dimorphic outcomes to immune challenge. Indeed, in the wild, environmental inputs come as combined, covariate packages, and it is in the context of these combined inputs that *Drosophila* has evolved. Higher-order, systemic signaling pathways such as IIS/mTOR may integrate these inputs to produce phenotypically plastic responses that match current environmental states (196). IIS/mTOR has been empirically demonstrated to regulate immune responses in *D. melanogaster* (198, 199), suggesting that IIS/mTOR could represent a nexus for integrating sex differences in responses to environmental variation, including immunity. Among the biotic factors that have gained much attention recently is the microbiome. Diet, of course, contributes to modeling the gut microbiome and its sex-specificity might shape dimorphic bacterial communities. Thus, microbiota-infection interactions (200, 201) have the potential to shape immune dimorphisms. Microbiome sex differences have been demonstrated in *Drosophila* (132), and likely interact with both dietary choices (133) and responses to infection, therefore it follows that a full understanding of immune dimorphisms cannot be achieved without considering natural poly-microbial interactions.

### The Ubiquitous Endosymbiont *Wolbachia* as a Troublemaker

*Wolbachia* is a ubiquitous endosymbiont predominantly transmitted by mothers to their offspring. This reproductive parasite is known to affect immune characteristics such as phenoloxidase (PO) activity (202), phagocytosis by hemocytes (203), and to affect the outcome of other infections, such as providing resistance to viral infection (204). While not the focus of the study, Martins et al. (60) showed that the level of resistance provided by *Wolbachia* status depends on the sex of the host. Furthermore, *Wolbachia* protects males more than females against enteric infection by the bacterium *P. aeruginosa* (205). For these reasons and because it is so common, we expect that *Wolbachia* infection could influence the direction of immune dimorphism, and even impact the evolution of the dimorphism itself.

### Regulation of Immune Dimorphism

Immune dimorphisms in *D. melanogaster* arise from a shared genome, and must be a product of sex-specific gene regulation, however, this is not yet well-resolved. At the top of the regulatory hierarchy are genomic differences, namely sex chromosome karyotype. As we have discussed, there are clear X- and Y-linked effects on immunity in *D. melanogaster*. Downstream of karyotype, dimorphisms can be regulated by the sex determination pathway, a cascade of splicing factors regulated by X chromosome number that ultimately lead to the expression of sex-specific transcription factors (206). This pathway has recently been shown to have “non-canonical” routes of signaling in some tissues (125), and importantly, to exert profound effects on physiology in larvae and adults beyond its purely developmental role (20). However, the potential regulation of immunity by sex-specific pathways is as yet untested [but see (125) for transcriptional regulation in the intestinal epithelium].

*Drosophila*, and insects in general, demonstrate that sexual dimorphism can occur without the presence of sex steroids *per se*. Notably, insects produce the steroid hormones ecdysone and juvenile hormone (JH), and as discussed, these are important regulators of immune function. It is possible that hormonal and genetic regulation interact; for example, via sex differences in hormone production or receptor expression (207). One potential source for dimorphic hormone production are the gonads, which may exert influences on immune responses in closely opposed organs, as has recently been demonstrated for regulation of intestinal carbohydrate metabolism by the testes (124). The tractability for genetic manipulation of sex-specific transcription factors and hormone production/reception is a major advantage to using *D. melanogaster* to better understand sex-specific regulation of immune function.

## Conclusion

We understand relatively little about immune dimorphisms in *Drosophila*, despite their apparent prevalence and magnitude. A real gap in our knowledge is the physiological and mechanistic underpinnings of the male-female differences in survival to infection that have been widely reported. We know almost nothing about how sex dimorphisms are shaped over the life course, including during development, or the influence of dimorphic immunity on aging and vice versa. Dimorphisms may arise from fixed differences in gene expression and tissue function, or from more plastic mechanisms that respond to environmental variables, or both. One thing is certain: the interaction between sex and immunity is complex. Complexity does not lend itself to generalities, and therefore we must be cautious in stating rules about male or female responses. Sex is clearly an essential factor that must be considered in the interpretation of data arising from studies into immunity, where the ideal approach is to include both sexes wherever possible. We argue that not only will including both sexes in studies of *Drosophila* immunity give a more complete picture, it will offer valuable insight into fundamental mechanisms underpinning innate immunity and responses to infection, and an understanding of the factors that drive dimorphisms to arise.

## Author Contributions

RB, M-KC, DD, and JR researched and wrote the manuscript. DD and JR planned and edited the manuscript. RB and M-KC performed the literature survey and made the figures, graphs and tables.

### Conflict of Interest

The authors declare that the research was conducted in the absence of any commercial or financial relationships that could be construed as a potential conflict of interest.

## Supplementary Material

Figure S1 Quantity of articles from “both” category with possible tag interactions. Total articles per tag are shown next to each tag title. If a tag is applied, it is indicated with a black dot. Black lines connecting dots indicates conditions where two or more tags were applied to the same article. Quantity of articles in each category are shown above the respective bar.

Table S1 Complete analysis of 5,626 articles retrieved from webofknowledge.com search. The Boolean search was: (TS = ((infection OR immunity OR hemocyte OR imd OR toll) AND drosophila) NOT TI = (bug OR bumblebee OR shrimp OR damesfly OR mollusc OR crab OR squid OR beetle OR baculovirus OR ant OR monochamus OR dastarcus OR cockroach OR crickets OR gryllus OR bemisia OR armyworm OR spodoptera OR mussel OR galleria OR helicoverpa OR amphibian OR manduca OR bee OR honey OR bactrocera OR tenebrio OR zebra OR dugesia OR flesh OR Apis OR house OR glossnia OR jelly OR Andrias OR dragonfly OR pachydiplax OR termite OR leech OR stick OR rhynchophorus OR rhodnius OR pardosa OR plutella OR coleoptera OR zophobas OR glossina OR ceratitis OR suzukii OR diabrotica OR rootworm OR sheep OR whitefly OR bird OR branchiostoma OR lizards OR laodelphax OR ceratopogonidae OR crassostrea OR oyster OR artemia OR freshwater OR calliphoridae OR phytomonas OR acyrthosiphon OR aphid OR crustacean OR parhyale OR hippocampus OR seahorse OR anopheles OR protaetia OR sea OR litopenaeus OR copepod OR swine OR planthopper OR arabidopsis OR circulifer OR leafhopper OR apostichopus OR cryptolaemus OR clam OR paphia OR mollusk OR achaea OR castor OR musca OR salmon OR dog OR echinococcus OR hetaerina OR sarcophaga OR fleshfly OR bovine OR zygoptera OR calopterygidae OR coenagrionidae OR scorpion OR locusta OR harpalus OR culex OR scylla OR firefly OR honeybees OR antheraea OR penaeus OR trichinella OR prawn OR macrobrachium OR ostrinia OR arge OR magnaporthe OR Phaeotabanus OR palm OR ostrinia OR daphnia OR scallop OR Chlamys OR Biomphalaria OR pig OR Anostostomatidae OR Orthoptera OR crayfish OR procambarus OR Platynereis)).
