# Supplementary figures and images for: Sexual Dimorphisms in Innate Immunity and Responses to Infection in Drosophila melanogaster
Source: Front Immunol. 2020 Jan 31;10:3075. doi: 10.3389/fimmu.2019.03075 (PMC7006818; doi:10.3389/fimmu.2019.03075)

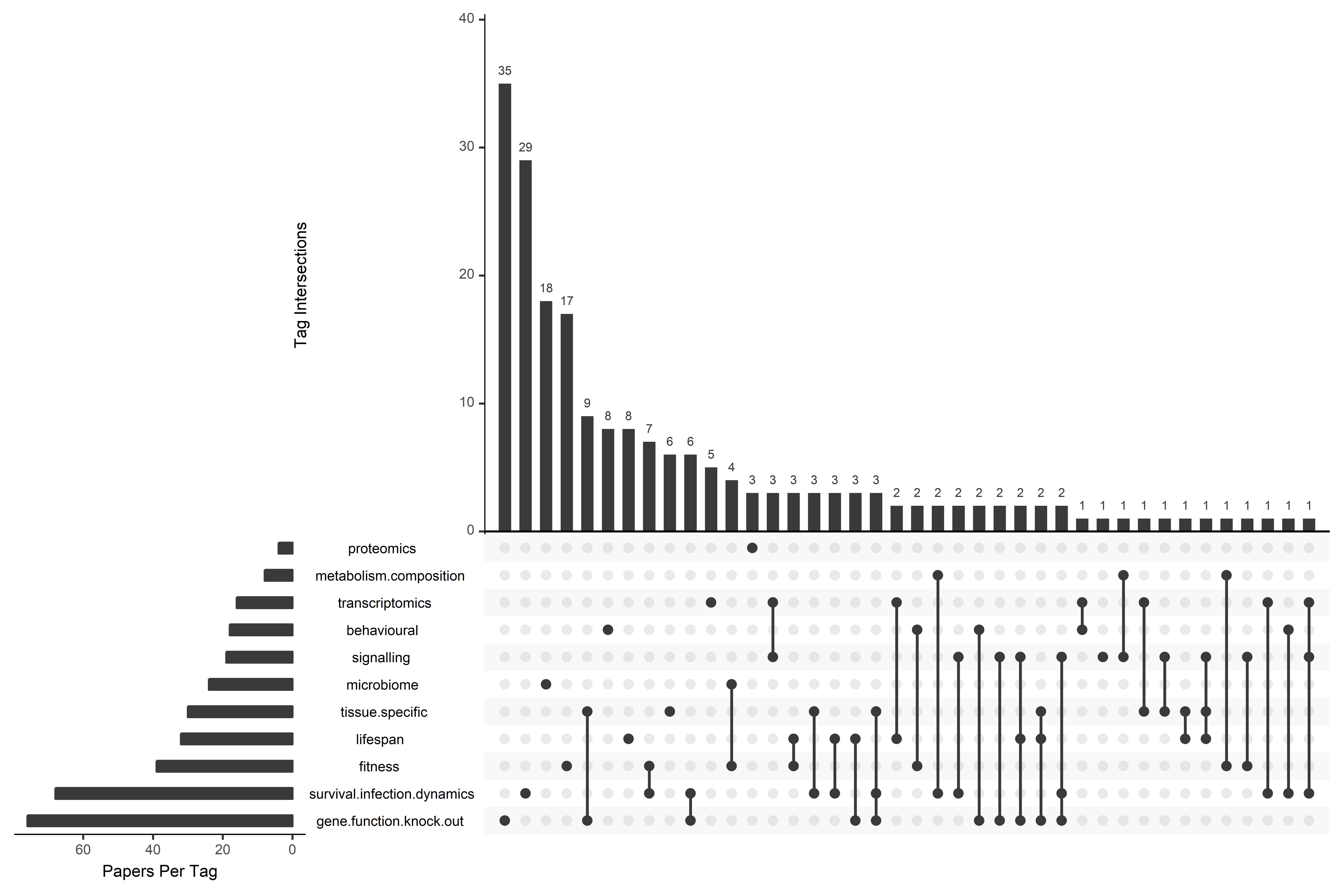

Supplement: Figure S1 — Quantity of articles from “both” category with possible tag interactions. Total articles per tag are shown next to each tag title. If a tag is applied, it is indicated with a black dot. Black lines connecting dots indicates conditions where two or more tags were applied to the same article. Quantity of articles in each category are shown above the respective bar. [file Image_1.PNG]
